# Supplementary material for: SMURF2 phosphorylation at Thr249 modifies glioma stemness and tumorigenicity by regulating TGF-β receptor stability
Source: Commun Biol. 2022 Jan 11;5:22. doi: 10.1038/s42003-021-02950-0 (PMC8752672; doi:10.1038/s42003-021-02950-0)
Supplement: Supplementary file 2 — Supplementary Information [file 42003_2021_2950_MOESM2_ESM.pdf]

Supplementary Table 1. List of oligonucleotides used for generation of shRNA.

Gene	Up (5'-3')	Down (5'-3')
sh*SMURF2* #1	GATCCGCCCACACTTGCTTCAATCTTCAA GAGAGATTGAAGCAAGTGTGGGCTTTTTTG	AATTCAAAAAAGCCCACACTTGCTTCAA TCTCTCTTGAAGATTGAAGCAAGTGTGGGCG
sh*SMURF2* #2	GATCCGATGAGAACACTCCAATTATTCAA GAGATAATTGGAGTGTTCTCATCTTTTTTG	AATTCAAAAAAGATGAGAACACTCCAATT ATCTCTTGAATAATTGGAGTGTTCTCATCG

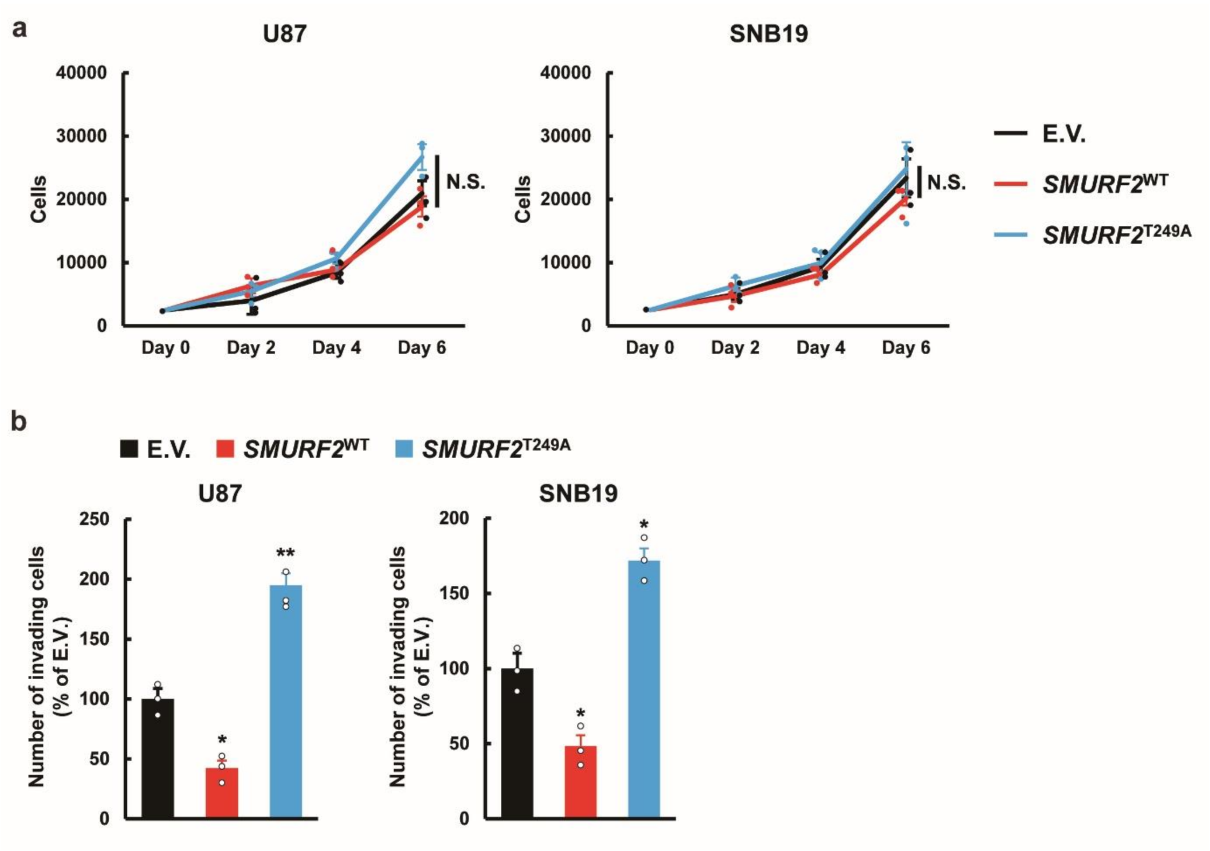

Supplementary Figure 1. SMURF2$^{\mathbf{Thr249}}$ phosphorylation regulates cellular function of differentiated glioma cells. U87 and SNB19 glioma cells were infected with *SMURF2*^{WT} or *SMURF2*$^{\text{T249A}}$, followed by determination of (a) cell number (n=3) and (b) invasive ability by transwell assay (n=3). *$P < 0.05$, **$P < 0.01$, significantly different from the value obtained in cells infected with E.V.. N.S., not significant. Values are expressed as the mean ± S.E. and statistical significance was determined using (a) two-way ANOVA *post hoc* Bonferroni test, and (b) one-way ANOVA *post hoc* Bonferroni test.

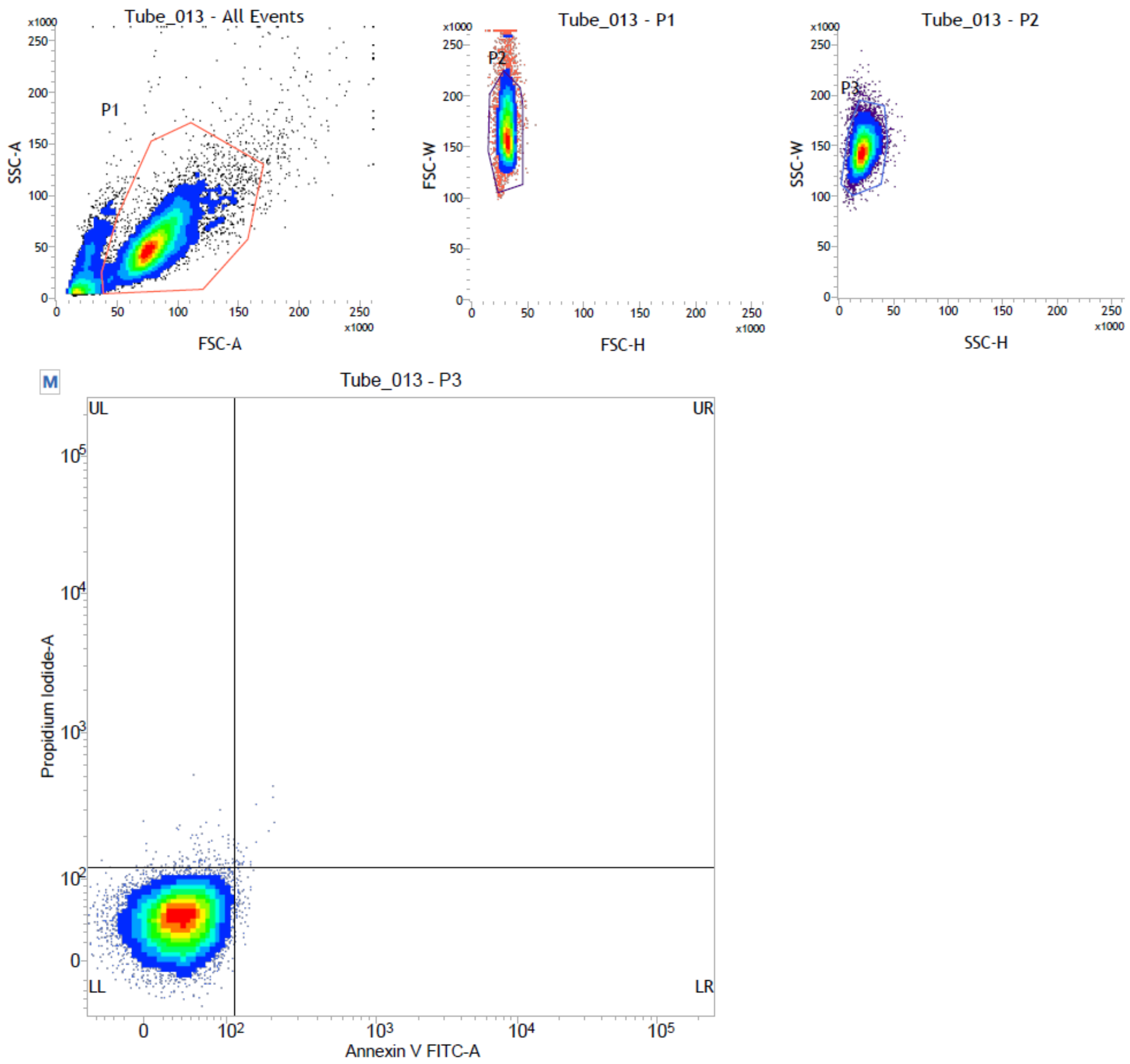

Supplementary Figure 2. Gating Strategy for Figure 1e and Figure 3e. Apoptosis assay was conducted using FITC-Annexin V Apoptosis Detection kit and analyzed by BD FACS Verse and BD FACSuite software. All cells were first gated on FSC/SSC according to cell size and granularity. FSC and SSC were used for identification of cell of interest and to exclude doublets. Unstained samples were used to set up negative gates. The gated cells were analyzed by FITC-Annexin V and Propidium Iodide fluorescence.

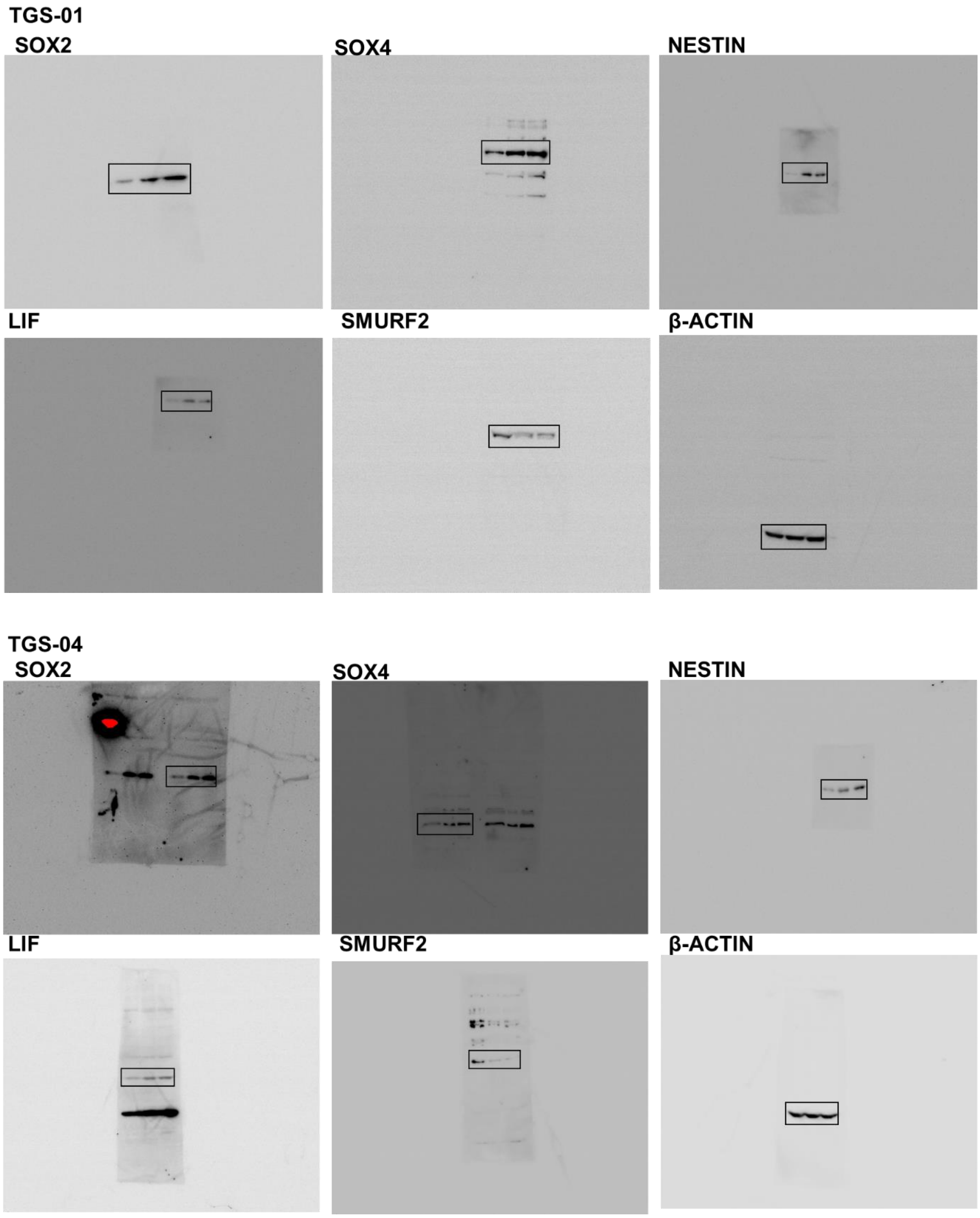

Supplementary Figure 3. Uncropped images of the membranes used for immunodetection shown in Figure 1c. TGS-01 and TGS-04 GSCs were infected with sh*SMURF2* (#1 and #2), followed by determination of protein levels of SOX2, SOX4, NESTIN, LIF, and SMURF2; β-ACTIN served as a loading control.

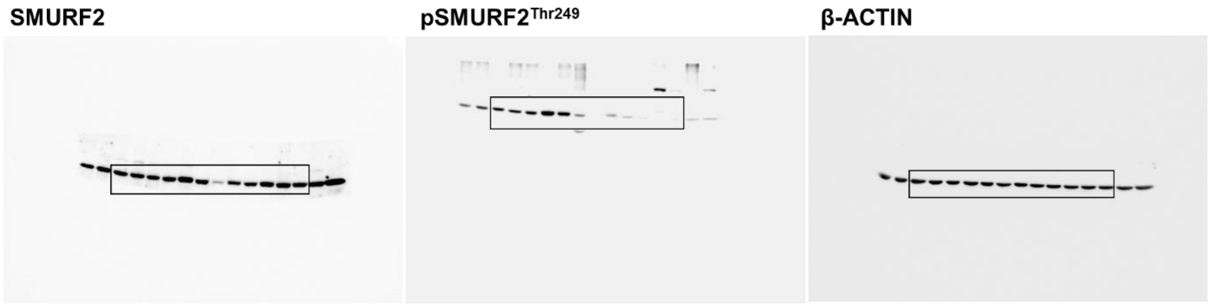

Supplementary Figure 4. Uncropped images of the membranes used for immunodetection shown in Figure 2b. Determination of protein levels of SMURF2 and pSMURF2$^{\text{Thr249}}$ in human glioma samples. Nonneoplastic brain tissue (NB), diffuse astrocytoma (DA) Grade II, anaplastic astrocytoma (AA) Grade III, glioblastoma (GBM) Grade IV.

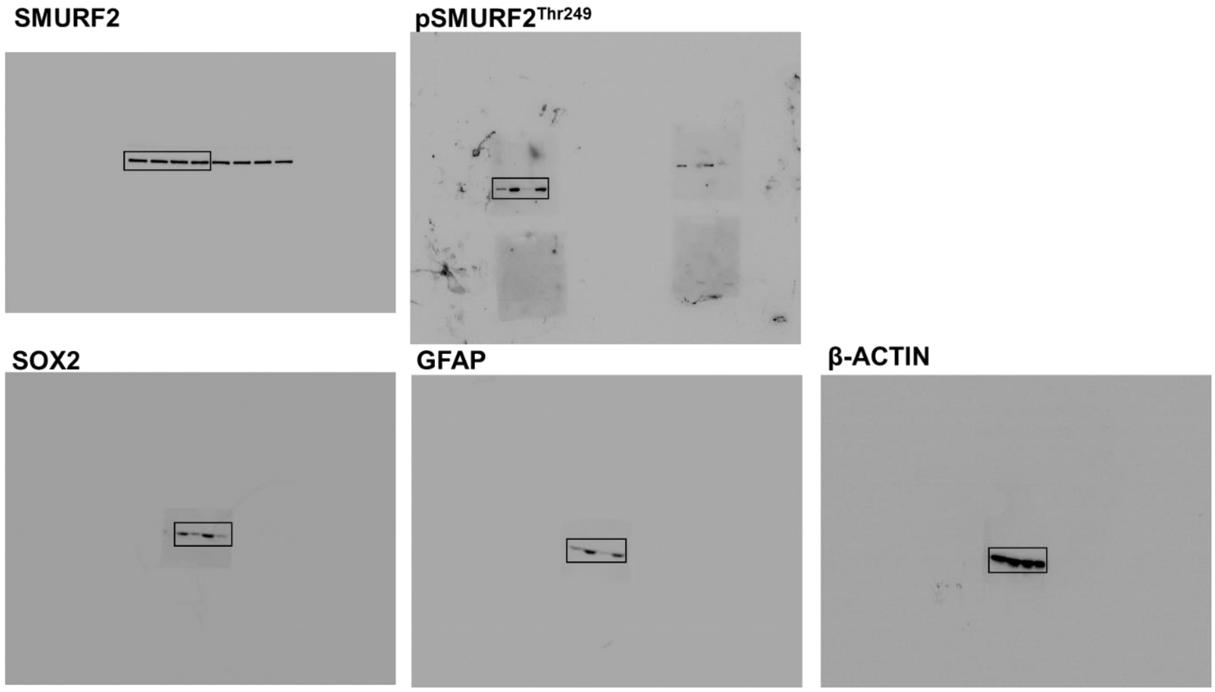

Supplementary Figure 5. Uncropped images of the membranes used for immunodetection shown in Figure 2f. TGS-01 and TGS-04 cells were cultured in neurosphere medium or adherent culture medium, followed by determination of protein levels of SMURF2, pSMURF2$^{\text{Thr249}}$, SOX2 and GFAP; β-ACTIN served as a loading control.

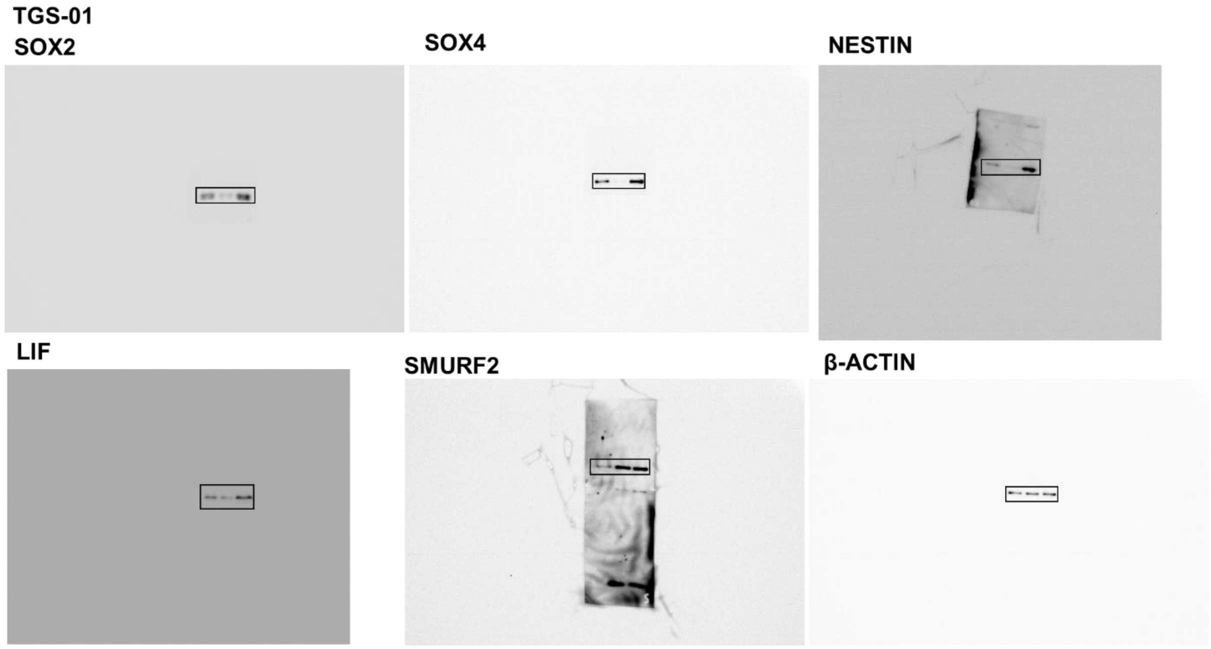

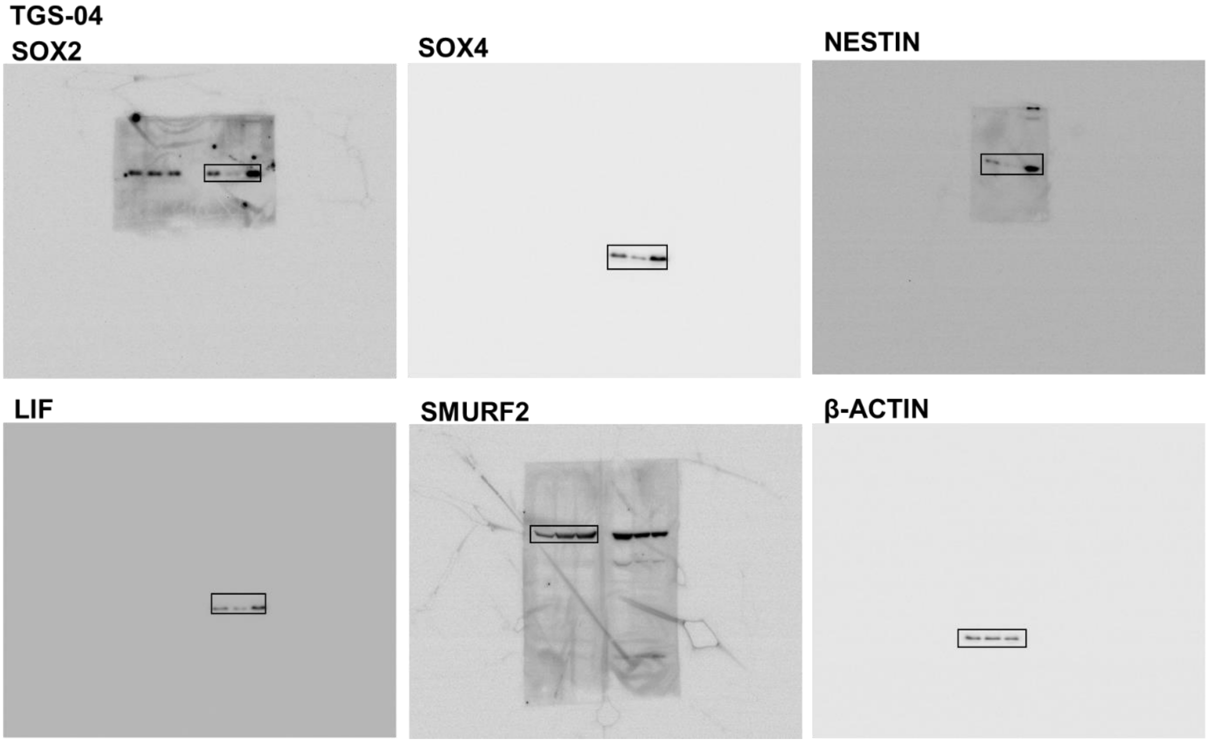

Supplementary Figure 6. Uncropped images of the membranes used for immunodetection shown in Figure 3c. TGS-01 and TGS-04 GSCs were infected with *SMURF2*^{WT} or *SMURF2*^{T249A}, followed by determination of protein levels of SOX2, SOX4, NESTIN, LIF, and SMURF2.

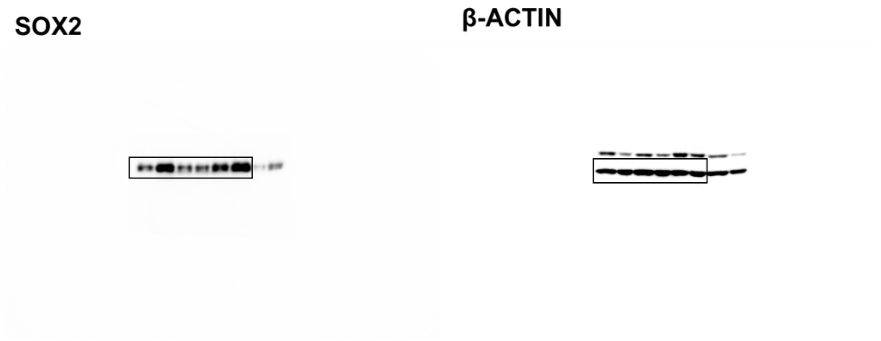

Supplementary Figure 7. Uncropped images of the membranes used for immunodetection shown in Figure 3h. Determination of protein levels of SOX2 in the brain of ipsilateral (Ipsi.) side of inoculation and contralateral (Cont.) side at 40 days after intracranial transplantation; β-ACTIN served as a loading control.

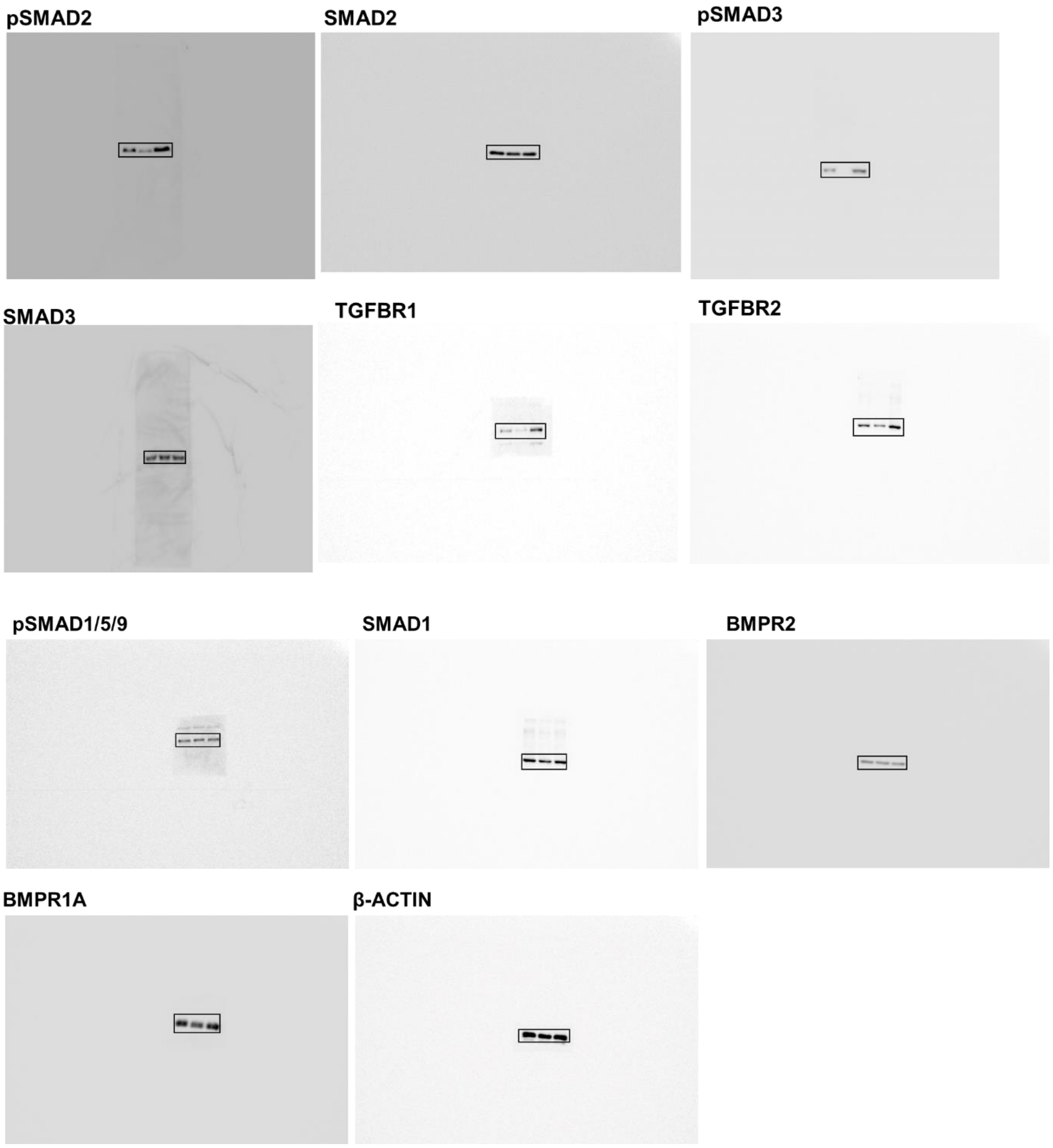

Supplementary Figure 8. Uncropped images of the membranes used for immunodetection shown in Figure 4a. TGS-01 GSCs were infected with *SMURF2*^{WT} or *SMURF2*^{T249A}, followed by determination of protein levels by immunoblotting.

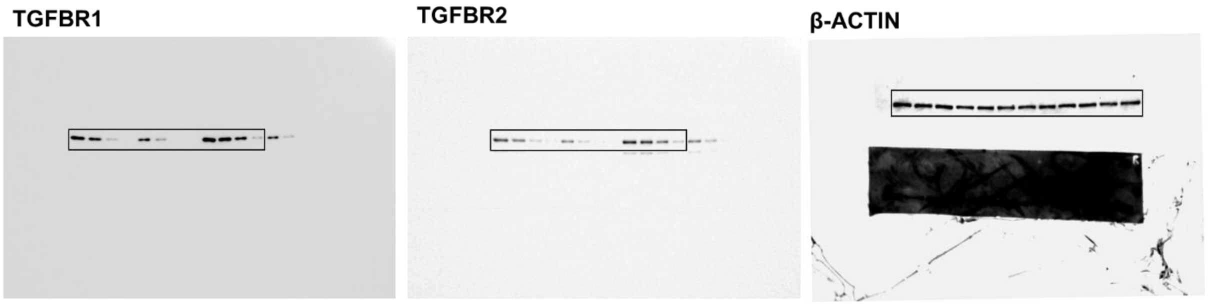

Supplementary Figure 9. Uncropped images of the membranes used for immunodetection shown in Figure 4b. TGS-01 GSCs were infected with *SMURF2*^{WT} or *SMURF2*^{T249A}, and treated with cycloheximide (CHX) at 50 µg/ml for indicated hours, followed by immunoblotting.

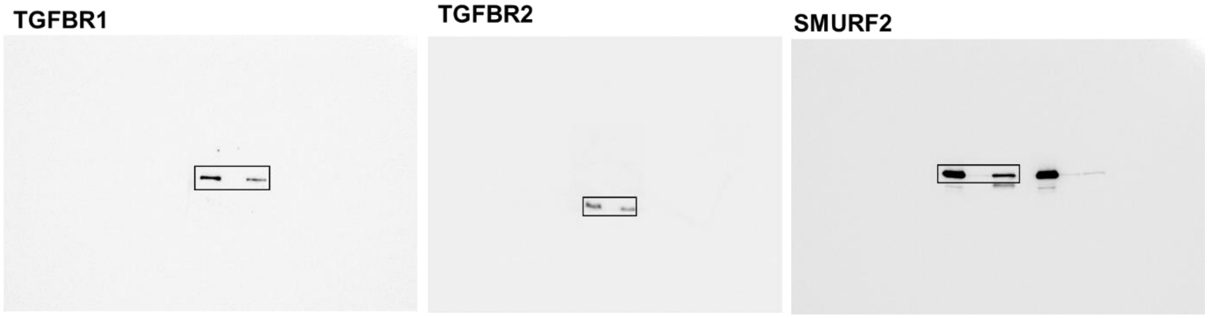

Supplementary Figure 10. Uncropped images of the membranes used for immunodetection shown in Figure 4c. Immunoprecipitation assay was performed in TGS-01 GSCs, followed by determination of protein levels of TGFBR1, TGFBR2 and SMURF2 by immunoblotting.

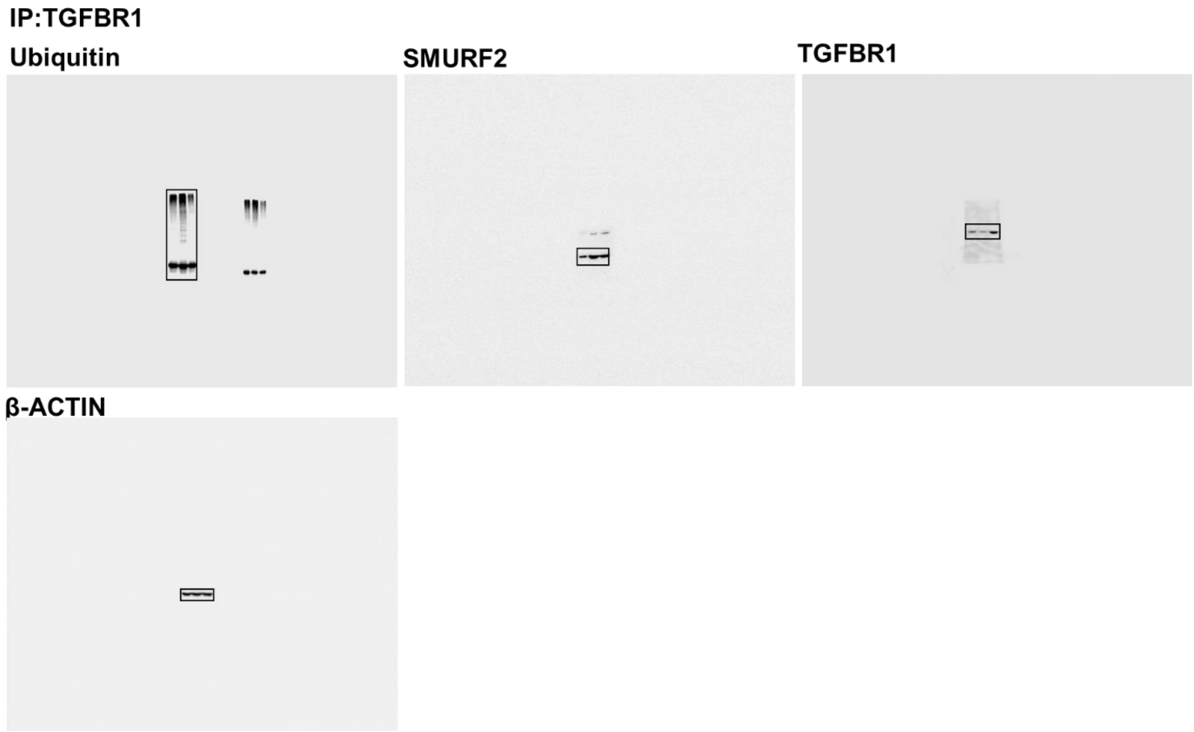

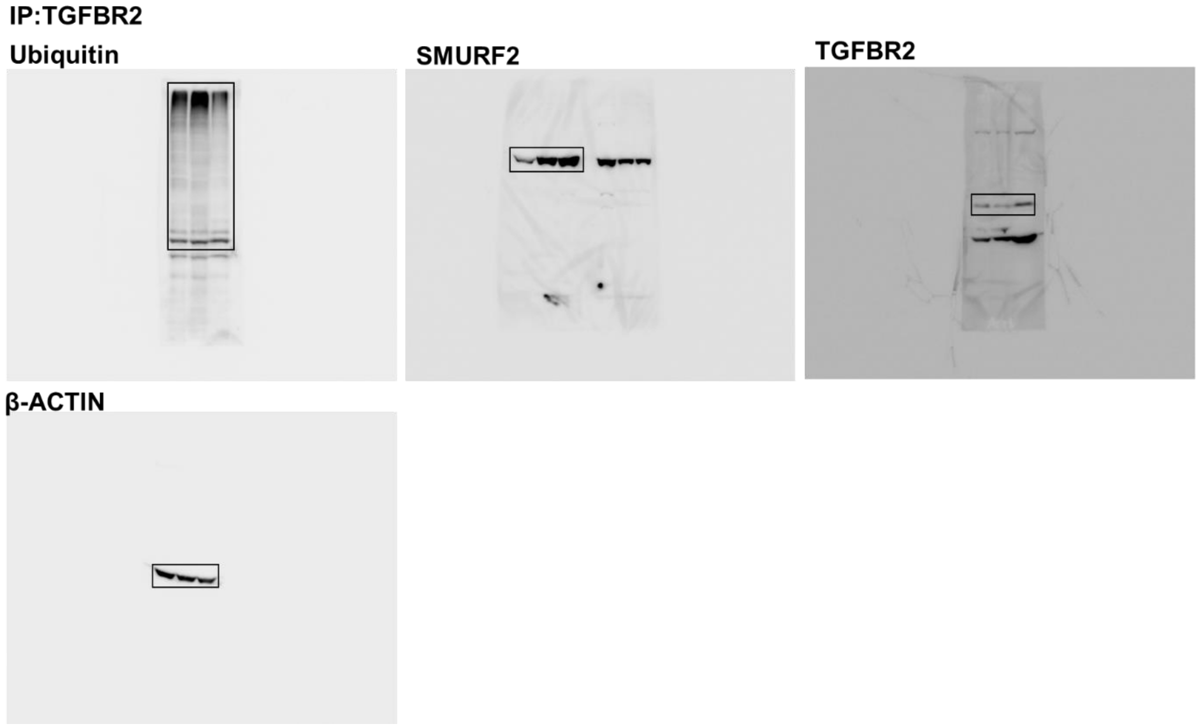

Supplementary Figure 11. Uncropped images of the membranes used for immunodetection shown in Figure 4d. TGS-01 GSCs were infected with *SMURF2*^{WT} or *SMURF2*^{T249A}, and subsequent immunoprecipitation with anti-TGFBR1 antibody or anti-TGFBR2 antibody, followed by determination of Ubiquitin with anti-Ubiquitin antibody.
