# Supplementary material for: SMURF2 phosphorylation at Thr249 modifies glioma stemness and tumorigenicity by regulating TGF-β receptor stability
Source: Commun Biol. 2022 Jan 11;5:22. doi: 10.1038/s42003-021-02950-0 (PMC8752672; doi:10.1038/s42003-021-02950-0)
Supplement: Supplementary file 3 — Description of Additional Supplementary Files [file 42003_2021_2950_MOESM3_ESM.pdf]

Description of Additional Supplementary Files

File name: Supplementary Data 1.
Description: Source data for all plots.
