# Supplementary material for: SMURF2 phosphorylation at Thr249 modifies glioma stemness and tumorigenicity by regulating TGF-β receptor stability
Source: Commun Biol. 2022 Jan 11;5:22. doi: 10.1038/s42003-021-02950-0 (PMC8752672; doi:10.1038/s42003-021-02950-0)
Supplement: Supplementary file 5 — Reporting Summary [file 42003_2021_2950_MOESM5_ESM.pdf]

nature research

Corresponding author(s): Takashi Iezaki and Eiichi Hinoi

Last updated by author(s): Nov 6, 2021

Reporting Summary

Nature Research wishes to improve the reproducibility of the work that we publish. This form provides structure for consistency and transparency in reporting. For further information on Nature Research policies, see our Editorial Policies and the Editorial Policy Checklist.

Statistics

For all statistical analyses, confirm that the following items are present in the figure legend, table legend, main text, or Methods section.

n/a	Confirmed	
☐	☒	The exact sample size (*n*) for each experimental group/condition, given as a discrete number and unit of measurement
☐	☒	A statement on whether measurements were taken from distinct samples or whether the same sample was measured repeatedly
☐	☒	The statistical test(s) used AND whether they are one- or two-sided<br>*Only common tests should be described solely by name; describe more complex techniques in the Methods section.*
☐	☒	A description of all covariates tested
☐	☒	A description of any assumptions or corrections, such as tests of normality and adjustment for multiple comparisons
☐	☒	A full description of the statistical parameters including central tendency (e.g. means) or other basic estimates (e.g. regression coefficient) AND variation (e.g. standard deviation) or associated estimates of uncertainty (e.g. confidence intervals)
☐	☒	For null hypothesis testing, the test statistic (e.g. *F*, *t*, *r*) with confidence intervals, effect sizes, degrees of freedom and *P* value noted<br>*Give P values as exact values whenever suitable.*
☒	☐	For Bayesian analysis, information on the choice of priors and Markov chain Monte Carlo settings
☒	☐	For hierarchical and complex designs, identification of the appropriate level for tests and full reporting of outcomes
☐	☒	Estimates of effect sizes (e.g. Cohen's *d*, Pearson's *r*), indicating how they were calculated

Our web collection on statistics for biologists contains articles on many of the points above.

Software and code

Policy information about availability of computer code

Data collection	Fluorescence microscope (Keyence, #BZ-X810) FACSVerse (BD Bioscience) and ChemiDoc Touch Imaging System (Bio-Rad)
Data analysis	Microsoft Excel 2019, ImageJ, FACSuite (BD Bioscience) and BZ-X800 Analyzer (Keyence)

For manuscripts utilizing custom algorithms or software that are central to the research but not yet described in published literature, software must be made available to editors and reviewers. We strongly encourage code deposition in a community repository (e.g. GitHub). See the Nature Research guidelines for submitting code & software for further information.

Data

Policy information about availability of data

All manuscripts must include a data availability statement. This statement should provide the following information, where applicable:

- Accession codes, unique identifiers, or web links for publicly available datasets
- A list of figures that have associated raw data
- A description of any restrictions on data availability

The bioinformatic data that support the findings of this study are openly available in GlioVis database (http://gliovis.bioinfo.cnio.es/).

Field-specific reporting

Life sciences study design

All studies must disclose on these points even when the disclosure is negative.

Sample size	No statistical methods were used to predetermine sample size. The sample sizes were based on previous studies using similar experiments.
Data exclusions	No data are excluded from the analyses.
Replication	All data were replicated at least once unless otherwise noted. The number of biological replicates (n) is indicated for each experiment.
Randomization	We randomly allocated the mice with matched age and sex in this study.
Blinding	The investigators were blinded to allocation during outcome assessment.

Reporting for specific materials, systems and methods

We require information from authors about some types of materials, experimental systems and methods used in many studies. Here, indicate whether each material, system or method listed is relevant to your study. If you are not sure if a list item applies to your research, read the appropriate section before selecting a response.

Materials & experimental systems

n/a	Involved in the study	
☐	☒	Antibodies
☐	☒	Eukaryotic cell lines
☒	☐	Palaeontology and archaeology
☐	☒	Animals and other organisms
☐	☒	Human research participants
☒	☐	Clinical data
☒	☐	Dual use research of concern

Methods

n/a	Involved in the study	
☒	☐	ChIP-seq
☐	☒	Flow cytometry
☒	☐	MRI-based neuroimaging

Antibodies

Antibodies used	Antibody used for Immunoblotting:<br>The primary antibodies used were, anti-p-Smurf2Thr249 (#J1683BA260-5, 1:2000) (GenScript), anti-Phospho-Smad2 (Ser465/467) (#3101, 1:1000), anti-Smad2 (#5339, 1:1000), anti-Phospho-Smad3 (Ser423/425) (#9520, 1:1000), anti-Smad3 (#9523, 1:1000), anti-TGF-β Receptor I (#3712, 1:1000), anti- TGF-β Receptor II (#79424, 1:1000), anti-Sox2 (#3579, 1:1000), anti-p-Smad1 (Ser463/465)/5 (Ser463/465)/9 (Ser465/467) (#13820, 1:1000), anti-Smad1 (#9512, 1:1000), anti-BMPR2 (#6979, 1:1000) and anti-Ubiquitin (#3936, 1:1000) (Cell Signaling Technologies), anti-β-actin (#sc-47778, 1:2000) (Santa Cruz Biotechnology), anti-Sox4 (#AB5803, 1:1000) (EMD Millipore), anti-NESTIN (#ab105389, 1:1000), anti-LIF (#ab138002, 1:1000), anti-BMPR1A (#ab174815, 1:1000) and anti-SMURF2 (#ab94483, 1:1000) (Abcam).<br>The secondary antibodies used were Anti-rabbit IgG, HRP-linked Antibody (#7074, 1:4000) and Anti-mouse IgG, HRP-linked Antibody (#7076, 1:4000) (Cell Signaling Technologies).<br><br>Antibody used for Immunoprecipitation:<br>The primary antibodies used were, anti-SMURF2 (#ab94483, 1:100) (Abcam), anti-TGF-β Receptor I (#3712, 1:100) and anti- TGF-β Receptor II (#79424, 1:100) (Cell Signaling Technologies).
Validation	All antibodies are validated by the manufacturers and by extensive use in published work.

Eukaryotic cell lines

Policy information about cell lines

Cell line source(s)	The human embryonic kidney 293 (HEK-293) T cells were obtained from RIKEN BRC. Human glioma cell lines U87 and SNB-19 were purchased from ATCC. TGS-01/04 were obtained from Cancer and Stem Cell Research Program, Division of Molecular Genetics, Cancer Research Institute, Kanazawa University.
Authentication	Cell lines were authenticated by the supplier. The use of TGS-01/04 and protocols were approved by the Ethics Committees of Gifu Pharmaceutical University, Kanazawa University, and the University of Tokyo.
Mycoplasma contamination	Cell lines were tested negative of mycoplasma contamination.
Commonly misidentified lines<br>(See ICLAC register)	n/a

Animals and other organisms

Policy information about studies involving animals; ARRIVE guidelines recommended for reporting animal research

Laboratory animals	4-week-old female nude mice (BALB/cSlc-nu/nu, SLC, Shizuoka, Japan)
Wild animals	n/a
Field-collected samples	n/a
Ethics oversight	All animal experiments were approved by the Committees on Animal Experimentation of Gifu Pharmaceutical University and Kanazawa University and performed in accordance with the guidelines for the care and use of laboratory animals.

Note that full information on the approval of the study protocol must also be provided in the manuscript.

Human research participants

Policy information about studies involving human research participants

Population characteristics	A total of 46 primary glioma tissues were obtained from patients who underwent surgical removal of tumor. The specimens were reviewed and classified according to WHO criteria. Nonneoplastic healthy brain tissues adjacent to tumors were acquired. Nonneoplastic brain tissue (NB) (n=12), diffuse astrocytoma (DA) Grade II (n=9), anaplastic astrocytoma (AA) Grade III (n=9), glioblastoma (GBM) Grade IV (n=16). Age, race, tumor location, presence of metastatsis, were not taken into consideration in choosing patients for the study.
Recruitment	The Glioma tissues were collected at the Department of Neurosurgery, Kanazawa University Hospital.
Ethics oversight	All experiments were approved by the local Institutional Review Board of Kanazawa University (No. 2509) and all study participants provided written informed consent.

Note that full information on the approval of the study protocol must also be provided in the manuscript.

Flow Cytometry

Plots

Confirm that:

- [x] The axis labels state the marker and fluorochrome used (e.g. CD4-FITC).
- [x] The axis scales are clearly visible. Include numbers along axes only for bottom left plot of group (a 'group' is an analysis of identical markers).
- [x] All plots are contour plots with outliers or pseudocolor plots.
- [x] A numerical value for number of cells or percentage (with statistics) is provided.

Methodology

Sample preparation	Cells were dissociated into single cells with StemPro Accutase (Gibco). Apoptosis assay was conducted using FITC-Annexin V Apoptosis Detection kit (BD Biosciences, #556547)
Instrument	BD FACSVerse
Software	BD FACSuite
Cell population abundance	For this analysis, 10 thousand cells were collected per sample.
Gating strategy	All cells were first gated on FSC/SSC according to cell size and granularity. FSC and SSC were used for identification of cell of interest and to exclude doublets. Unstained samples were used to set up negative gates. The gated cells were analyzed by FITC-Annexin V and Propidium Iodide fluorescence.

- [x] Tick this box to confirm that a figure exemplifying the gating strategy is provided in the Supplementary Information.
